# Understanding gene regulatory mechanisms by integrating ChIP-seq and RNA-seq data: statistical solutions to biological problems

**DOI:** 10.3389/fcell.2014.00051

**Published:** 2014-09-17

**Authors:** Claudia Angelini, Valerio Costa

**Affiliations:** ^1^Istituto per le Applicazioni del Calcolo “M. Picone” - CNRNapoli, Italy; ^2^Computational and Biology Open Laboratory (ComBOlab)Napoli, Italy; ^3^Institute of Genetics and Biophysics “A. Buzzati-Traverso” - CNRNapoli, Italy

**Keywords:** ChIP-seq, data integration, gene regulatory mechanisms, RNA-seq, statistics

## Abstract

The availability of omic data produced from international consortia, as well as from worldwide laboratories, is offering the possibility both to answer long-standing questions in biomedicine/molecular biology and to formulate novel hypotheses to test. However, the impact of such data is not fully exploited due to a limited availability of multi-omic data integration tools and methods. In this paper, we discuss the interplay between gene expression and epigenetic markers/transcription factors. We show how integrating ChIP-seq and RNA-seq data can help to elucidate gene regulatory mechanisms. In particular, we discuss the two following questions: (i) Can transcription factor occupancies or histone modification data predict gene expression? (ii) Can ChIP-seq and RNA-seq data be used to infer gene regulatory networks? We propose potential directions for statistical data integration. We discuss the importance of incorporating underestimated aspects (such as alternative splicing and long-range chromatin interactions). We also highlight the lack of data benchmarks and the need to develop tools for data integration from a statistical viewpoint, designed in the spirit of reproducible research.

## Introduction

High-throughput technologies have made the collection of genome-wide data in cells, tissues and model organisms easier and cheaper. These data allow one to investigate biological aspects of cell functionality and to better understand previously unexplored disease etiologies. Nowadays, RNA-seq and ChIP-seq are widely used to measure gene expression and to obtain genome-wide maps of transcription factor (TF) occupancies and epigenetic signatures (Park, 2009; Wang et al., 2009; Costa et al., 2010; Ozsolak and Milos, 2011; Furey, 2012). Several computational tools have been developed to independently analyze these data, both for single sample characterization and differential analysis (Pepke et al., 2009; Garber et al., 2011; Bailey et al., 2013). The interplay between transcriptomics and epigenomics has been widely demonstrated. Chromatin accessibility to the transcription machinery regulates gene expression and, *viceversa*, some non-coding RNAs can affect local chromatin states (Wang et al., 2011b). Such interplay has significant biomedical implications in physiological processes and pathologic states (Feng et al., 2014). Therefore, integrating ChIP-seq and RNA-seq data is a compelling need to predict gene expression during cell differentiation and development (Comes et al., 2013; Lesch et al., 2013; Malouf et al., 2013; Jiang et al., 2014; Kadaja et al., 2014) and to study human diseases, including cancer (Portela and Esteller, 2010).

The seminal work of Hawkins et al. (2010) explained why integrative omic data analysis can provide unprecedented opportunities to address some long-standing questions about genome functions and diseases. To date, large-scale data produced by ENCODE/GENCODE (ENCODE Project Consortium., 2012; Harrow et al., 2012), Cancer Genome Atlas (http://cancergenome.nih.gov/), Roadmap Epigenomics (http://www.roadmapepigenomics.org) offer the possibility to answer specific questions, as well as to raise, formulate and test novel hypotheses and questions in life science. However, despite the pros, multi-omic data integration is still one of the most challenging problems in modern science (Gomez-Cabrero et al., 2014).

**Figure 1:**
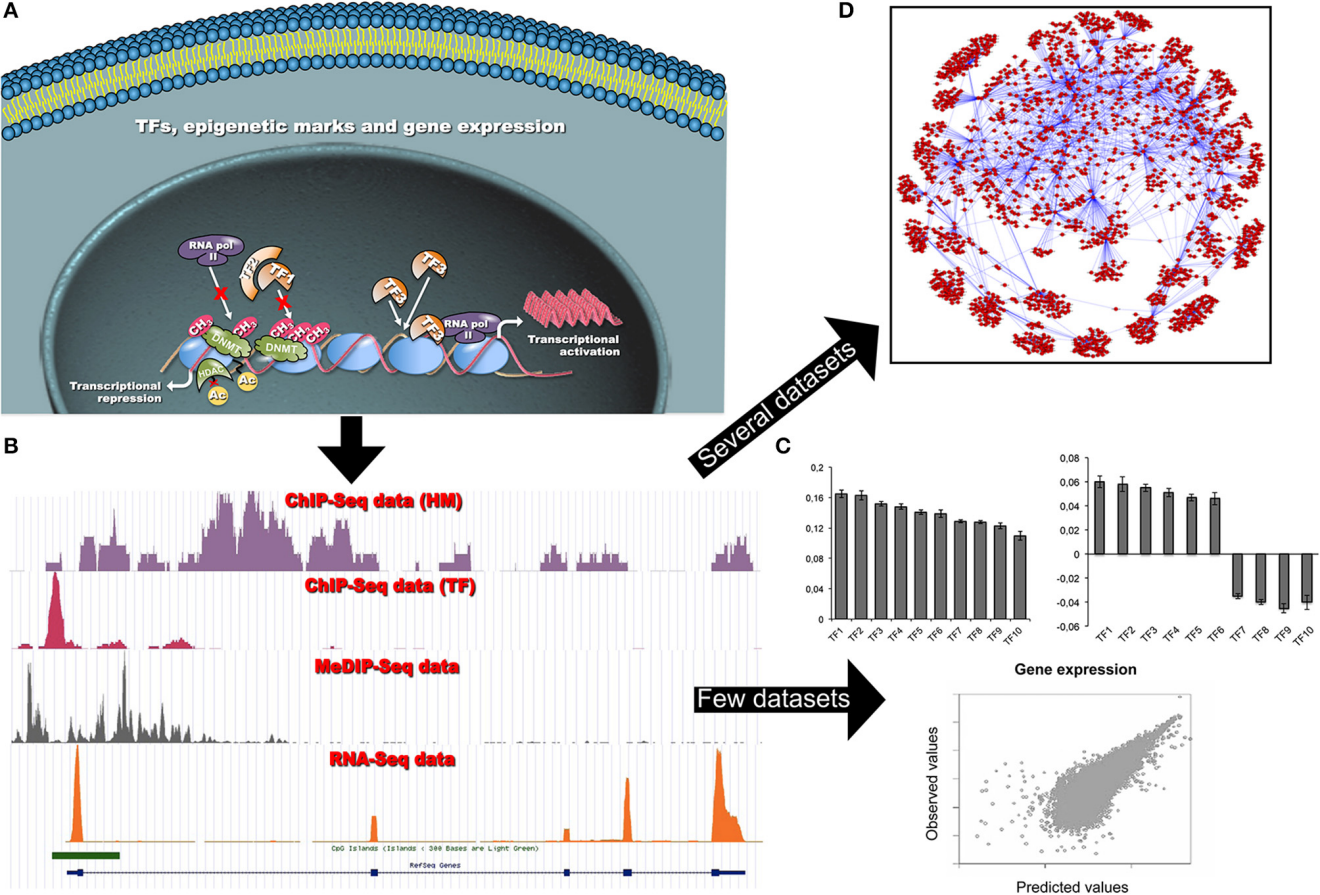
**(A)** Schematic representation of the dynamic interactions among chromatin modifications and TFs, and their impact on gene transcription in a cell. Different cells share the same TF binding sites despite of differences in functionality, shape and differentiation state. Transcriptional patterns are controlled by differential TF bindings and other factors, such as local chromatin states and epigenetic modifications. These factors can limit, or promote, TF occupancies at specific *loci*, and regulate gene transcription. **(B)** Each NGS coverage data track (bedgraph format) is representative of the result of a single omic data analysis (i.e., ChIP-seq or RNA-seq experiments). The visualization of several tracks allows qualitatively studying a specific gene locus. The computational analysis of single omics allows investigating (on a genome-wide scale) different epigenetic modifications (TFs, HMs, CpG methylation, chromatin accessibility) and measuring gene expression. **(C)** When a limited amount of ChIP-seq (TF binding and/or HMs) and RNA-seq datasets are available, simple predictive models based on PCA and log-linear or support vector regression are used to predict gene expression and to reveal the most relevant epigenetic signatures able to explain the gene expression. By plotting loading factors it is possible to reveal that epigenetic signatures can act either as activators or repressors of transcription at different loci (see Section Can TF occupancies or histone modification data predict gene expression?). **(D)** In the presence of a large number of gene expression datasets more sophisticated models can be used to infer complex GRNs. This network allows visualizing TF-gene relations. In particular, it is possible to show that a given TF can control several genes and that genes are strongly interconnected (see Section Can ChIP-seq and RNA-seq data be used to infer gene regulatory networks?).

In this paper we discuss the following questions: (i) how to explain and predict gene expression (and differential expression) and (ii) how to define gene regulatory network (GRN) in humans or model organisms using epigenetic data (Figure 1). Section Gene regulation and its impact in biology and medicine describes the biological context. Section An overview on ChIP-seq and RNA-seq data integration approaches and tools contains an overview of data visualization and integration tools. Section Statistical solutions to some biological questions illustrates the most recent statistical advances for ChIP-seq and RNA-seq data integration. Finally, Section Open biological questions and future perspectives enlightens our perspective view on the open biological questions and the tools that need to be developed in the next years. Section Conclusions reports our conclusions.

## Gene regulation and its impact in biology and medicine

The sole nucleotide sequence of a gene does not explain its functions nor its regulation. Gene transcription is specified by DNA structure and by its accessibility to the basal transcription machinery. A physical interaction of TFs, chromatin-modifying enzymes (histone acetyl/methyltransferases and deacetylases/demethylases) and other accessory proteins with DNA is needed to modulate transcription dynamics, determining cell fate (Atkinson and Halfon, 2014). Local chromatin states and epigenetic modifications can limit, or promote, TF occupancies at specific *loci*. Several diseases can result from the alteration of chromatin remodeling and gene transcription (Portela and Esteller, 2010). Thus, understanding—and controlling—such processes may help to define potential therapies, as well as to drive cell differentiation toward specific directions.

Many efforts have been made to measure transcript levels, to detect differential expression and to identify novel alternatively spliced transcripts in various conditions (reviewed in Costa et al., 2010, 2013; Steijger et al., 2013; Angelini et al., 2014). However, regardless of the technology, a challenge is to explain and to predict gene expression by means of the coordinated binding of TFs, epigenetic marks and long-range interactions among distant chromatin domains. Recent studies demonstrate that the binding of specific TFs and some histone modifications (HMs) can be used to predict gene expression *in vitro* and to identify relevant epigenetic actors (Ouyang et al., 2009; Karlić et al., 2010; Cheng et al., 2011a, 2012; McLeay et al., 2012). Analogously, gene expression changes have been correlated to modification of TF bindings and chromatin marks (Althammer et al., 2012; Klein et al., 2014).

In general, gene expression can be predicted using a limited number of samples (in specific conditions). On the opposite, inferring large GRNs can be reached only using several high-throughput datasets, as in Gerstein et al. (2012). However, some networks can be less complicated than expected and can rely on a low number of factors and interactions. Dunn et al. (2014) recently identified a minimal set of components (12 TFs and 16 interactions) sufficient to explain the self-renewal of ES cells.

In terms of potential impact on human genetics, we highlight the following considerations. Cell differentiation is accompanied by global—and local—chromatin changes, leading to the silencing of pluripotency genes and lineage-specific gene activation (Chen and Dent, 2014). In this regard, multi-omic integration and single-cell omics can be used to explain and to potentially control differentiation and to explore heterogeneity of cells in development and disease (Comes et al., 2013; Macaulay and Voet, 2014).

Understanding such mechanisms will significantly improve the treatment of human genetic diseases, particularly of cancer. Indeed, epigenetic—unlike genetic—modifications are reversible, and modulating epi-marks through up/down-regulation of histone methyltransferases can affect gene expression and tissue-specific alternative splicing (Luco et al., 2010, 2011). By correcting the aberrant distribution of epi-marks, we may in turn control pathologic changes in gene expression (Schenk et al., 2012). In this regard, the proper identification of aberrant epigenetic regulators in tumors is of major interest. The final objective is to identify new therapeutic targets and to develop novel molecules (*epi-drugs*, inhibitors or activators of histone acetyl/methyltransferases and deacetylases/demethylases) that are able to correct or prevent aberrant epi-marks (Mai and Altucci, 2009). These interesting compounds promise to define more efficient cancer treatment strategies.

## An overview on ChIP-seq and RNA-seq data integration approaches and tools

Data integration can be achieved with different methodologies. Genome browsers and other multidimensional visualization tools (Schroeder et al., 2013) provide integrated environments to navigate and visualize heterogeneous experimental data. Multi-omic data visualization in few loci of interest helps to formulate novel functional hypotheses. However, this is not sufficient to fully benefit from the genome-wide information that next-generation sequencing (NGS) data can provide. Naive approaches, so far used to integrate epigenetic signatures with gene expression, annotate (by proximity) either peaks or enriched regions with genes. The epigenetic profiles are displayed on the top of the gene structures. Then enriched regions are associated to pathways and gene ontologies by means of gene names (McLean et al., 2010; Statham et al., 2010; Zhu et al., 2010; Lawrence et al., 2013).

Nowadays, public repositories represent a relevant data source. Few web-based resources provide integrated information at both epigenetic and transcriptional levels, e.g., ChIP-Array (Qin et al., 2011), EpiRegNet (Wang et al., 2011a), ISMARA (Balwierz et al., 2014), and GeneProf (Halbritter et al., 2011, 2014). In particular, the latter allows one retrieving data and results of already processed ChIP-seq and RNA-seq studies; each result is connected to the workflow used to generate it. Therefore, previous results can be easily integrated with user data. Other computational platforms, such as Galaxy (Goecks et al., 2010), constitute a general framework for omic data integration.

All these approaches are very useful to summarize and visualize global information or to identify associations among different data types. However, they do not provide mathematical models for explanatory and predictive inference, as methods described in Section Statistical solutions to some biological questions.

## Statistical solutions to some biological questions

The questions posed in Section Introduction and illustrated in Figure 1 are discussed in the next subsections.

### Can TF occupancies or histone modification data predict gene expression?

**Figure 2:**
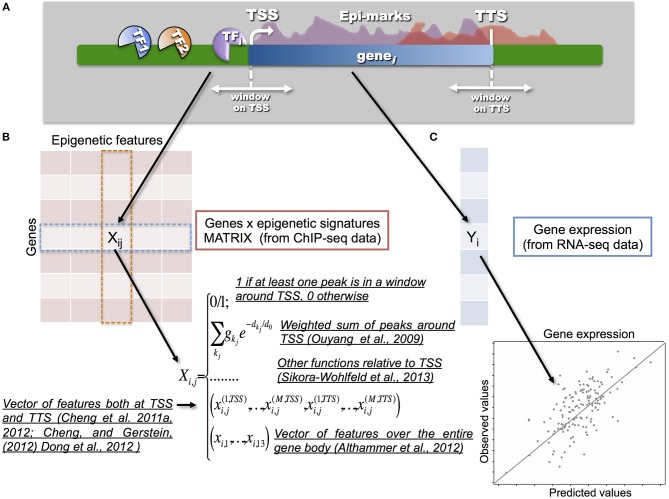
**One of the key-points in the integration process is the way in which the epigenetic and transcriptional signals are transformed into a statistical model that relates a response vector Y (i.e., gene expression) with a set of predictors, represented by a matrix X (i.e., epigenetic signatures). (A)** A scheme showing gene transcription, and the molecular factors involved (TFs and HMs), is illustrated in the upper part. **(B)** Different models have been proposed to build the so-called gene to epigenetic signature matrix **X**. Naive models proposed to use a binary matrix to integrate epigenetic signatures with gene expression. Therefore, 0/1 values were used to annotate and associate a given TF or HM to a specific gene according to a proximity measure between the peak and/or the enriched region and TSS of the corresponding gene. More advanced models, such as the one from Ouyang et al. (2009), proposed to use a weighed sum of peaks around the TSS. In this way it is possible to tune the strength of the binding and the distance from the TSS in a continuous way. Along the same direction, Sikora-Wohlfeld et al. (2013) compared several other measures to build **X**. All such approaches share the idea that matrix **X** is built with respect to the position of the TSSs (or using reads in a window around the TSSs) by collapsing each epigenetic feature into a single value per gene. A slightly different, and more sophisticated, approach consists in mapping each epigenetic feature into a vector of several components measured (in several bins) both at the TSSs and TTSs, as proposed in the series of papers by Cheng and colleagues. In this way, they showed that the best predictive power for TFs is indeed achieved at TSSs, however for HMs the information available at TTSs can provide further improvement. Finally, a set of 13 features for each epigenetic mark is used in Althammer et al. (2012) to classify genes as up-regulated; down regulated and no-change between two experimental conditions. The features are evaluated over the gene body, on its upstream and downstream regions (including promoters, TSSs, first exons, first introns, etc). **(C)** Gene expression Y (usually measured in terms of Fragment per kilobase of exon per million fragments mapped, FPKM) is obtained from RNA-seq data.

The work of Ouyang et al. (2009) represents one of the first attempts to address the question using ChIP-seq and RNA-seq data and log-linear regression. In this framework, gene expression is regarded as a response variable and different TF-related features as predictors. The authors build the TF association strength matrix **X** as a weighted sum of intensities of peaks surrounding the genes of interest (Figure 2). They found that a remarkably high proportion of gene expression variation can be explained by the binding of 12 specific TFs. Principal component analysis (PCA) revealed that these TFs may have a dual effect. They can activate a subset of genes and repress other ones. Similarly, a simple model selection regression strategy shows that gene expression can be accurately predicted using only a small number of HMs (Karlić et al., 2010). The combined usage of different epigenetic features and chromatin accessibility data (DNase I hypersensitive sites from DNase-seq), within a log-linear regression and PCA further improves gene expression prediction (McLeay et al., 2012). More interestingly, McLeay and colleagues demonstrated that *in silico* TF binding prediction could be used as surrogate information, in absence of *in vivo* binding data.

Differently, Cheng and co-authors (Cheng et al., 2011a, 2012; Cheng and Gerstein, 2012; Dong et al., 2012) mapped each epigenetic feature into a vector of several components, measured both at the transcription starting sites (TSSs) and at the transcription termination sites (TTSs). They showed that TF binding achieves the highest predictive power in a small region centered at the TSS, whereas HMs have high predictive power in wider regions across genes. Their approach differs both for the building of the feature matrix and for the use of support vector regression. The latter does not assume a linear relationship between gene expression and signals for TFs or HMs, allowing one to capture more complex relationships. Other supervised and unsupervised statistical methods have been proposed in Xu et al. (2010); Hebenstreit et al. (2011); Park and Nakai (2011); Gagliardi and Angelini (2013). The advantage of the above-described statistical approaches is that they allow carrying out both explanatory and predictive inference.

Previous methods focused on single biological systems for which both RNA-seq and ChIP-seq data are available. In principle, the same methods could be applied to correlate gene expression variations and changes in epigenetic mark densities between two conditions. In this context, Althammer et al. (2012) used 13 features for each epigenetic mark and a machine learning approach (based on random forest) to classify genes as up-, down-regulated or no-change when comparing two conditions. The vectors of features are extracted from TFs and HMs, and also DNase-seq and DNA methylation data. More recently, approaches based on Bayesian mixture models have been used to detect genes with differential expression and variations in the HM profiles between two experimental conditions (Klein et al., 2014).

Despite the differences in the statistical models, all the above-mentioned approaches revealed that it is possible to predict gene expression using genome-wide TF occupancies or HM data.

### Can ChIP-seq and RNA-seq data be used to infer gene regulatory networks?

The availability of several gene expression datasets generated from knock-out cells for one or few TFs has made possible to infer GRNs. Reconstructing GRNs using gene expression data has been one of the most widely studied problems in the last decade (Wang and Huang, 2014). However, the integration of TF occupancies data and mRNA expression values, as well as data from other transcriptional and post-transcriptional regulators, can improve methods for inferring GRNs. This task still constitutes a challenge in system biology especially for complex organisms.

ChIP-seq data were first used to determine target genes and miRNAs using data from modENCODE (Cheng et al., 2011b). Then, a regulatory network was obtained by using the correlation between TF binding and gene expression. A more comprehensive study, involving hundreds of TFs from ENCODE disclosed several structural properties of human regulatory networks (Gerstein et al., 2012). Both studies are mainly descriptive (i.e., analysis of how regulatory information is organized) and do not fully benefit from the amount of information available in terms of improving inferential approaches.

Under the assumption that network sparseness is higher in complex than in small genomes, GRN inference can be turned into a sparse optimization problem (LpRGNI, Qin et al., 2014). The identification of a small TF set that controls the network is obtained by solving a regularized lasso-type problem. The integration of ChIP-seq data improves the inference performance. As an alternative, as proposed in CMGRN (Guan et al., 2014), Bayesian network models can be first used to infer causal interrelationship among TFs and HMs (i.e., to understand how several regulators influence or associate with each other) by analyzing the sequences of regulators based on ChIP-seq read counts on the promoter of target genes. Then, Bayesian hierarchical Gibbs sampling allows integrating ChIP-based regulatory signals of TFs and HMs, microRNA binding targets with differential expression profile of genes, to construct GRN at different levels (epigenetic, transcriptional and post-transcriptional).

In general, we are far from inferring realistic quantitative models of genome-wide regulatory networks. However, it is possible to reveal the main interactions and the most relevant players. Then, computational methods can refine sub-networks for specific functions. In this spirit, Dunn et al. (2014) first generated all possible networks that could explain stem cell self-renewal. Then, by using formal verification procedures and Boolean network formalisms, they selected a core network of only 12 TFs and 16 interactions, showing that ES self-renewal relies on a relatively low number of factors and interactions.

## Open biological questions and future perspectives

From a biological perspective, data integration is not *an end* to answer fundamental questions, but *a means* to generate new hypotheses. In this regard, genome-wide omic data are fundamental to drive researchers into a deeper understanding of many biological aspects (Hawkins et al., 2010).

To date, there is a limited use of multi-omic data. The association between epigenetic features and genes is still mainly done according to their proximity with respect to TSSs (with few exceptions, Althammer et al., 2012) and the existing approaches only account for local interactions. Moreover, genome-wide maps (by ChIA-PET and Hi-C) of long-range chromatin interactions and of chromatin nuclear organization have not been fully integrated in the previously described inferential models. Regression approaches in Section Can TF occupancies or histone modification data predict gene expression? are based on assumption of independence between genes, whereas the physical proximity of genes in the chromosomes in the nucleus is evidence of physical interaction. Therefore, we suggest that future computational methods for multi-omic data integration include information from genome-wide long-range interaction studies. To this aim, we propose the use of locus-by-locus interaction matrix, as a kind of correlation matrix within a regression model.

Similarly, chromatin accessibility data (Thurman et al., 2012) such as DNase-seq data, DNA regions associated with regulatory activity (FAIRE-seq), and DNA methylation data (MeDip-seq and BS-seq) should be used to better model DNA-binding background and reduce the number of false positive relations (as also suggested by Cheng et al., 2012). In such cases, we believe that the approaches described by Althammer et al. (2012) could be useful. However, the choice of the initial set of features has to be tuned according to the specific omic data at hand. Then, feature selection strategies have to be applied.

In absence of *in vivo* data, surrogate data (based on computational predictions or data from closely related cell lines or conditions) could be used to decrease experimental costs. McLeay et al. (2012) and Liò et al. (2012) showed in two different contexts that such strategy is feasible and can improve the results. Further studies should be devoted to investigate pros and cons of such approaches.

Another interesting consideration comes from the evidence that relatively few factors (TFs and/or HMs) are sufficient to explain gene expression quite accurately. Such an apparent redundancy for HMs (Cheng and Gerstein, 2012) opens the question whether such factors have a causal function or only constitute a regulatory code. Notably, such redundancy has been described only with regard to gene expression levels, without taking into account alternative splicing and differential isoform abundance. We hypothesize that the observed redundancy could partially account for a different layer of complexity, poorly explored till now. Many recent evidences indicate that some epi-marks are associated to tissue-specific alternative splicing (Luco et al., 2010, 2011; Ye et al., 2014). In this regard, the works from Chen and Dent (2014) have tried to partially overcome this issue by achieving higher predictive accuracy. Although this approach led to a higher predictive accuracy, it was not able to capture the differential expression of transcripts sharing the same TSS. We believe that a more sophisticated analysis may reveal that different combinations of epigenetic patterns can tune isoform switching (e.g., controlling the type of alternative splicing) and determine their relative abundance. The answer to such a complex question is still a challenge.

We want to underline that, despite the possibility to predict gene expression using few epigenetic features, no causal relationships can be directly inferred from such methods. The possibility of determining whether causal relationships exist or markers only constitute a code (Henikoff and Shilatifard, 2011; Cheng and Gerstein, 2012) requires developing causal inference that till now received only limited attention (Yu et al., 2008; Guan et al., 2014). In this regard, we propose Bayesian models to carry on causal inference.

Finally, while there exist several tools for data visualization (as described in Section An overview on ChIP-seq and RNA-seq data integration approaches and tools), only few tools implementing the statistical algorithms (Section Statistical solutions to some biological questions) are available. In addition, there are not general tools that allow comparing the developed methods for gene expression prediction and GRN on the same benchmarks. In light of these considerations, it is now very difficult for biologists to carry on data integration. Therefore, to facilitate biologists in such a task we strongly emphasize the need to develop new and intuitive explorative tools for the integration of ChIP-seq and RNA-seq data from a statistical viewpoint. Moreover, we firmly believe such tools should be designed in the spirit of reproducible research (Goecks et al., 2010; Russo and Angelini, 2014) to allow reproducibility and transparent verification of published results and to improve transfer of knowledge.

## Conclusions

The diffusion of high-throughput technologies has offered the possibility to answer new questions, but has also posed new challenges to old problems in life science, such as data integration (Gomez-Cabrero et al., 2014). Indeed, data integration is gradually losing the merely descriptive function (as representation of data from different sources) and it is quickly acquiring inferential role. In this *scenario*, statistical methods can be used not only to analyze specific types of omic data, but also to integrate them within explanatory and predictive models. Such models can be used for further inference and to simulate the effect of specific changes *in silico*. However, to fully exploit the data available from international consortia, novel statistical methods and tools are required. In this paper, we discussed the work carried out in the last few years, and we provided our perspective about future developments.

### Conflict of interest statement

The authors declare that the research was conducted in the absence of any commercial or financial relationships that could be construed as a potential conflict of interest.
